# Anti-Photoaging Effects of Soy Isoflavone Extract (Aglycone and Acetylglucoside Form) from Soybean Cake

**DOI:** 10.3390/ijms11124782

**Published:** 2010-11-24

**Authors:** Chieh-Chen Huang, Bo-Yang Hsu, Nan-Lin Wu, Wen-Huei Tsui, Tzu-Ju Lin, Ching-Chieh Su, Chi-Feng Hung

**Affiliations:** 1 School of Medicine, Fu Jen Catholic University, Taipei, Taiwan; E-Mail: 054317@mail.fju.edu.tw; 2 Department of Dermatology, Shin Kong Wu Ho-Su Memorial Hospital, Taipei, Taiwan; E-Mail: m001017@ms.skh.org.tw; 3 Department of Food Science, Fu Jen Catholic University, Taipei, Taiwan; E-Mail: alginate1001@yahoo.com.tw; 4 Department of Dermatology, Mackay Memorial Hospital, Hsinchu, Taiwan; E-Mail: d94443002@ntu.edu.tw; 5 Mackay Medicine, Nursing and Management College, Taipei, Taiwan; 6 Department of Life Science, Fu Jen Catholic University, Taipei, Taiwan; E-Mail: 002381@mail.fju.edu.tw; 7 Graduate Institute of Basic Medicine, Fu-Jen Catholic University, Taipei, Taiwan; E-Mail: 054317@gmail.com; 8 Division of Endocrinology and Metabolism, Department of Internal Medicine, Cardinal Tien Hospital, Taipei, Taiwan; E-Mail: suk.ccsu@msa.hinet.net

**Keywords:** isoflavone, aglycone, acetylglucoside, UVB, catalase, COX-2

## Abstract

Soy isoflavones, found in soybean and soybean products, have been reported to possess many physiological activities such as antioxidant activity, inhibition of cancer cell proliferation, reduction of cardiovascular risk, prevention of osteoporosis and alleviation of postmenopausal syndrome. In our previous study, soy isoflavone extract ISO-1 (containing 12 soy isoflavones) from soybean cake was demonstrated to prevent skin damage caused by UVB exposure. In this study, soy isoflavone extract from soybean cake was further purified and evaluated for the protective effects on UVB-induced damage. The results revealed that Fraction 3, which contains the aglycone group (daidzein, genistein and glycitein) and acetylglucoside group (acetyldaidzin, acetylgenistin and acetylglycitin) of soy isoflavones, could inhibit UVB-induced death of human keratinocytes and reduce the level of desquamation, transepidermal water loss (TEWL), erythema and epidermal thickness in mouse skin. Furthermore, topical application of Fraction 3 increased the activity of catalase and suppressed cyclooxygenase-2 (COX-2) and proliferating cell nuclear antigen (PCNA) expression in mice exposed to UVB. In addition, in comparison with ISO-1 and genistein, the Fraction 3 possessed much greater protective effects on both UVB-induced oxidative stress and keratinocyte death than other fractions. Therefore, the soy isoflavone extract Fraction 3 from soybean cake is a desirable anti-photoaging agent for skin care.

## 1. Introduction

Ultraviolet (UV) radiation, especially UVB (280–320 nm) from sunlight, is one of the major environmental hazards to induce skin damage. UV exposure could cause edema, erythema, hyperpigmentation, hyperplasia, photoaging, inflammation, DNA damage and mutations in the skin [1,2]. Moreover, an epidemiologic study reported that long-term exposure to UV radiation raised the risk of skin cancer [3]. Another study revealed that mitogen-activated protein kinase (MAPK) and p38 were activated by UV exposure, followed by increased expression of cyclooxygenase-2 (COX-2), which contributed to cutaneous inflammation and even carcinogenesis [4]. Furthermore, reactive oxygen species (ROS), such as H_2_O_2,_ could be induced by UV exposure and this led to oxidative stress and skin cell damage [5]. In an animal study, acute and chronic UV exposure caused acute sunburn and skin aging in hairless mice [6].

Soy isoflavones contain 12 different isoforms and can be divided into four chemical forms: aglycone (daidzein, genistein and glycitein), glucoside (daidzin, genistin and glycitin), acetylglucoside (acetyldaidzin, acetylgenistin and acetylglycitin), and malonylglucoside (malonyldaidzin, malonylgenistin and malonylglycitin). Soy isoflavones have been demonstrated to possess many biological functions such as antioxidant [7], inhibition of cancer cell proliferation [8], anti-inflammatory activities [9], prevention of coronary heart diseases [10], as well as osteoporosis [11]. In anti-inflammatory effects, soy isoflavones could decrease the secretions of interleukin-1 (IL-1), IL-6, nitric oxide (NO) and prostaglandin E_2_ (PGE_2_) in the cell supernatant and fluid of mouse peritoneal exudate [8].

Soybean cake is a byproduct during the processing of soybean oil and contains large amount of soy isoflavones. Four isoflavone groups from soybean cake, namely aglycone, glucoside, malonylglucoside and acetylglucoside, have been purified and have been found to possess differential antioxidant activities [7]. The acetylglucoside exhibits the highest efficiency in 1,1-diphenyl-2-picrylhyhrazyl (DPPH) scavenging assay and the glucoside has the highest efficiency in chelating metal ions. This study also indicated that the aglycone and acetylglucoside groups possess better antioxidant activities than other soy isoflavone groups [7]. Recently, we have demonstrated that the UVB protective effects of soy isoflavones might be related to their antioxidant activities [5]. Moreover, soy isoflavone extract inhibited UVB-induced keratinocyte death and suppressed UVB-induced intracellular H_2_O_2_ release, which reduced oxidative stress. Furthermore, it also decreased the epidermal thickness and inhibited COX-2 and proliferating cell nuclear antigen (PCNA) expression. UVB-triggered activation of p38, c-Jun N-terminal kinase (JNK) and extracellular signal regulated kinase (ERK1/2) were inhibited by treatment with soy isoflavone extract [5,12,13]. Therefore, the aims of this study were to prepare Fraction 3 from soybean cake, which contains the aglycone group (daidzein, genistein and glycitein) and acetylglucoside group (acetyldaidzin, acetylgenistin and acetylglycitin) of soy isoflavones, and to evaluate the anti-photoaging effects.

## 2. Results and Discussion

### 2.1. Soy Isoflavone Extract Fraction 3 Inhibits UVB-Induced Keratinocyte Death

**Figure 1.**
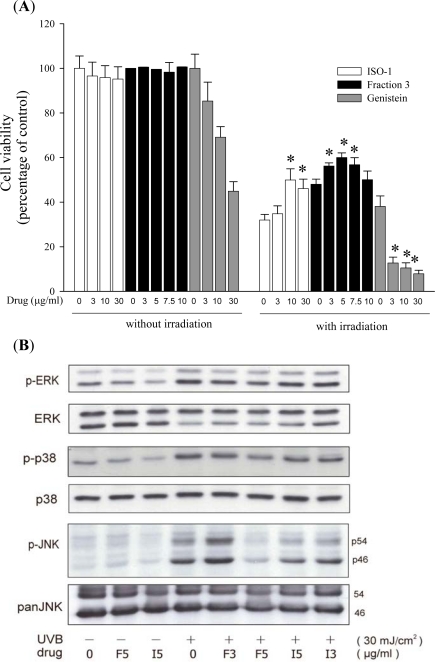
(**A**) Fraction 3 was not cytotoxic to keratinocytes, (□) ISO-1; (▪) Fraction 3; (▪) genistein. UVB irradiation-induced cell death decreased after treatment with ISO-1 and Fraction 3 by MTT assay. Results in (A) are expressed as percentage of control and the mean ± S.E. (n = 3). *P < 0.05 *vs.* control. (**B**) Effects of Fraction 3 and ISO-1 on UVB-induced ERK1/2, p38 and JNK phosphorylation. HaCaT cells were preincubated with Fraction 3 and ISO-1 for 24 h, followed by UVB irradiation. After incubation, cells were collected and lysates were analyzed by Western blot analysis, (F) Fraction 3; (I) ISO-1.

Cell viability was 95–100% when treated with the soy isoflavone extract Fraction 3 (Fraction 3), which contains both the aglycone and acetylglucoside forms of isoflavones. The result indicated that soy isoflavone extract Fraction 3 from soy cake is non-toxic for human keratinocytes. In order to explore the photoprotective effects of Fraction 3 on keratinocytes, cells were treated with Fraction 3 (3, 5, 7.5, 10 μg/mL). In control cells, the cell viability significantly decreased after UVB exposure. However, treatment of cells with Fraction 3 obviously increased the cell viability. With the optimal concentration, 5 μg/mL, the cell viability increased about 12% (compare with the control UVB exposure group). The protective effects had no significant difference between 3 and 7.5 μg/mL, and thus the results suggest that Fraction 3 still had a protective effect at a lower concentration (3 μg/mL). In contrast, soy isoflavone extract ISO-1 (containing 12 soy isoflavones) had no significant protective effects on UVB-induced keratinocyte death at the concentration of 3 μg/mL. Moreover, genistein decreased the cell viability in a dose-dependent manner (Figure 1A). In Western blot analysis, the levels of phosphorylation of ERK1/2, p38 and JNK in cells treated with Fraction 3 (3 and 5 μg/mL) were lower than those treated with ISO-1 (3 and 5 μg/mL; Figure 1B). Therefore, Fraction 3 possessed more protective effects on UVB-induced keratinocyte death than ISO-1 and genistein.

Several studies indicated that MAPK could be activated by UVB exposure, which was related to cell damage [12–14]. In addition, reactive oxygen species (ROS), especially H_2_O_2_, act as mediators to activate the MAPK pathway after UVB exposure [12,15]. Three MAPK signal pathways have been identified, including ERK1/2, JNK and p38. The activation of these kinases correlated with skin cell damage in response to UVB exposure [5,12–15]. Besides, the sustained p38 activation by UVB exposure in turn led to cytochrome c release and procaspase-3 activation [5,16]. Moreover, JNK mediates UV-induced apoptosis via the mitochondrial pathway [5,17].

Chiang *et al* (2007) reported that the four groups of isoflavone extract from soybean cake significantly increase cell viability and inhibit intracellular H_2_O_2_ production [5]. The acetylglucoside form of soy isoflavones possessed the highest protective efficiency against UVB-induced cell death. Moreover, another study demonstrated that the acetylglucoside form of isoflavones possesses relatively stronger antioxidant activity [7]. Given that ROS act as mediators to regulate MAPK pathways (ERK1/2, p38 and JNK), suppression of ROS production might lead to inhibition of MAPK pathways. Therefore, we suggest that the protective effects of Fraction 3 against UVB-induced keratinocyte death might be attributed to their antioxidant activity.

### 2.2. Soy Isoflavone Extract Fraction 3 Attenuates the Level of Erythema and TEWL after UVB Exposure

**Figure 2.**
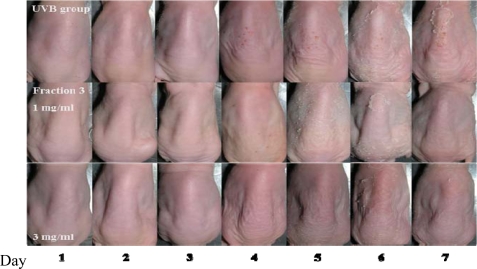
Photoprotective effects of Fraction 3 on UVB-induced pathological changes in the skin surface. Nude mice were treated with UVB irradiation in the presence or absence of Fraction 3 for seven days. One and three mg/ml of Fraction 3 was applied onto nude mice before UVB irradiation. Days after vehicle and Fraction treatment are shown.

**Figure 3.**
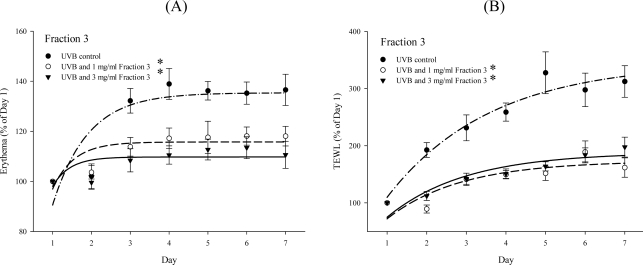
Change in physiology parameters on the skin surface after treatment with Fraction 3. (**A**) Change in erythema after treatment with Fraction 3. (**B**) Change in transepidermal water loss (TEWL) after treatment with Fraction 3.

UVB exposure causes transepidermal water loss (TEWL) and erythema of the skin [12]. Next, the level of wrinkles and desquamation in UVB-exposed skin was examined in the presence or absence of Fraction 3, and it was increased at 4–7 days during seven-days of UVB irradiation as compared with non-exposed skin. However, treatment with Fraction 3 (1 and 3 mg/mL) significantly decreased the wrinkles and desquamation in the dorsal skin of mice (Figure 2). The level of erythema and TEWL was also evaluated in the skin after seven days UVB exposure. Again, treatment with Fraction 3 (1 and 3 mg/mL) obviously suppressed the level of erythema and TEWL. There was no significant difference between the 1 and 3 mg/mL groups (Figure 3A and B). However, the blood flow and melanin in the dorsal skin of mice showed no significant difference between the control group and Fraction 3 group (data not shown). Kim *et al.* [18] indicated that mice fed with food rich in soy isoflavones for four weeks had a lower level of UVB-induced wrinkles in the skin. In addition, UVB exposure caused collagen degradation, which contributed to increase the level of wrinkles in the skin. Another study also revealed that the destruction of collagen fibers in the dermis contributed to UV-induced photoaging. The expression of matrix metalloproteinase 1 (MMP-1) by UV exposure is the primary cause of the collagen destruction [19]. The UVB-induced desquamation also enhanced the level of TEWL and wrinkles in the skin. In this study, before UVB exposure, treatment with Fraction 3 significantly decreased the level of desquamation in the dorsal skin of mice and consequently reduced the level of TEWL and wrinkles. UVB also induced inflammation caused erythema of the skin. However, before UVB exposure, treatment with Fraction 3 could diminish the level of erythema. Because soy isoflavone extract had been demonstrated to possess anti-inflammatory activities [8], we suggest that the effect on decreasing the level of erythema might be attributed to their anti-inflammatory activities.

### 2.3. Soy Isoflavone Extract Fraction 3 Reduces the Epidermal Thickness

**Figure 4.**
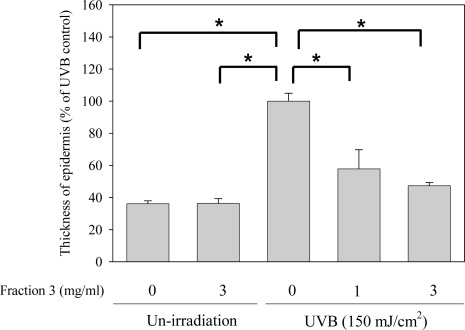
Fraction 3 inhibits UVB-induced epidermal proliferation, *p < 0.05.

The epidermis growth factor receptor (EGFR) can be activated by UVB exposure leading to an increase of the thickness of the epidermis [20]. In our results, the epidermal thickness of the dorsal skin of mice of the control group was 13.08 ± 0.65 μm and of the UVB exposure group was 36.20 ± 1.79 μm. However, the epidermal thickness was significantly diminished by treatment with Fraction 3 and the average epidermal thickness was 20.94 ± 4.32 (1 mg/mL) and 17.14 ± 0.70 (3 mg/mL). Compared with the UVB exposure group, treatment with 1 and 3 mg/mL of Fraction 3 could reduce the epidermal thickness by about 43 and 53%, respectively (Figure 4). The EGFR has been reported to promote epidermal hyperplasia and the epidermal thickness in normal mice was higher than that of mice lacking EGFR after UVB exposure [20]. According to our results, the reduced epidermal thickness by Fraction 3 might be related to its interference with EGFR expression.

### 2.4. Soy Isoflavone Extract Fraction 3 Decreases the Depletion of Catalase after UVB Exposure

**Figure 5.**
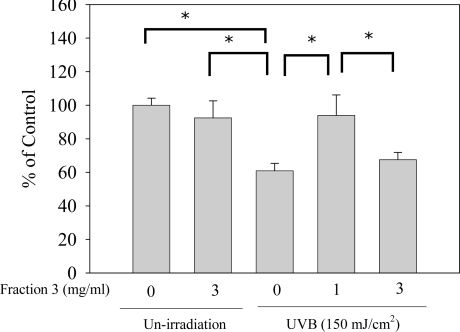
Effects of Fraction 3 on catalase activity. Data is shown as mean ± S.E. (n = 4). *p < 0.05 compared to the control group.

UVB exposure could elicit H_2_O_2_ production. Nevertheless, H_2_O_2_ was converted to oxygen and water by catalase so UVB exposure might contribute to the decrease of the catalase activity [12]. Moreover, catalase is able to reduce oxidative stress. In our results, after UVB exposure, catalase was significantly depleted in the dorsal skin of ICR-Foxn/^nu^ mice. Compared with the non-exposure group, the catalase activity was reduced by about 40% in the UVB exposure group. However, treatment with Fraction 3 (1 and 3 mg/mL) significantly enhanced the catalase activity and there was no notable difference between 1 mg/mL and the non-exposure group (Figure 5). In addition, compared with our recent study, when treated with 1 mg/mL of Fraction 3, the average activity of catalase (92%) was higher than with treatment with 1 mg/mL of ISO-1 (85%). In addition, treatment with genistein could not increase the catalase activity [12]. Hence, the results suggested that Fraction 3 possesses the highest efficiency in reducing oxidative stress. Chiang *et al*. [5] pointed out that soy isoflavone extract could inhibit UVB-induced H_2_O_2_ production in keratinocytes. Consequently, we suggest that the effect of increasing catalase activity of Fraction 3 might be attributed to diminution of intracellular H_2_O_2_ production.

### 2.5. Soy Isoflavone Extract Fraction 3 Suppresses the Expression of COX-2 and PCNA after UVB Exposure

**Figure 6.**
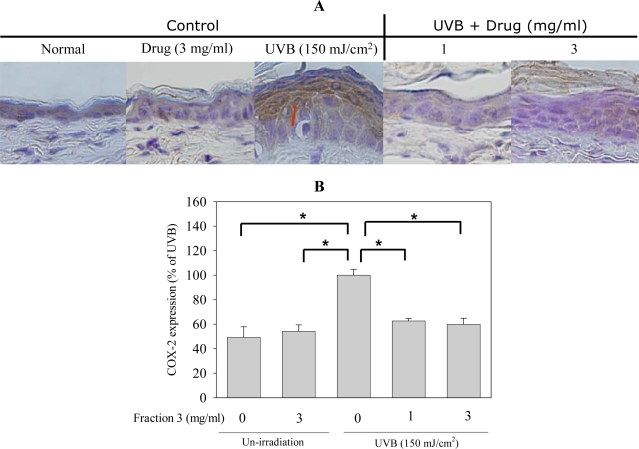
Fraction 3 inhibits UVB irradiation increased COX-2 expression. (**A**) COX-2 expression by immunohistochemistry, magnification 400 X. (**B**) Quantification of COX-2 expression by counting positively staining cells, mean ± S.E. (n = 4). *p < 0.05 compared to the control group.

Cyclooxygenase (COX) is the critical enzyme in the biosynthesis of the prostaglandins (PGs) modulating inflammation and other important physiological processes. COX has two major isoforms, COX-1 and COX-2. COX-1 is a housekeeping enzyme, is constitutively expressed in most tissues and mediates normal physiological functions (e.g., platelet aggregation, regulation of renal blood flow). COX-2 is inducible and can be induced by multiple mitogenic and inflammatory stimuli, such as hormones, growth factors, cytokines, tumor promoters and UV light. The physiological functions mediated by COX-2 include fever, inflammation, pain, vasodilation, angiogenesis and increased vascular permeability [21,22]. UVB exposure could induce COX-2 expression, which was regulated by the signal transduction pathways including the activation of tyrosine kinase, p38 MAPK or Akt [23–26]. Furthermore, COX-2 expression in the skin had been demonstrated to result in the induction of edema, epidermal hyperplasia, inflammation, and skin carcinogenesis [22]. Next, we examined whether Fraction 3 affected COX-2 expression in mice exposed to UVB. The COX-2 expression was observed in epidermal cells of the dorsal skin of mice after UVB exposure, as determined by immunohistochemistry. Topical application with isoflavone extract Fraction 3 (1 and 3 mg/mL) before UVB exposure clearly decreased COX-2 expression. Compared with the non treatment group, the expression of COX-2 decreased by about 40% after treatment with Fraction 3 (1 and 3 mg/mL; Figure 6A and B). Several studies indicated that ROS such as H_2_O_2_ might play an important role in UVB-induced COX-2 expression and pretreatment with the antioxidant partly inhibited the UVB-induced COX-2 expression in HaCaT cells [26]. Therefore, we suggest that the inhibition of COX-2 expression by Fraction 3 might result from its antioxidant activity and anti-activation of UVB-induced ERK1/2, JNK and p38 MAPK.

**Figure 7.**
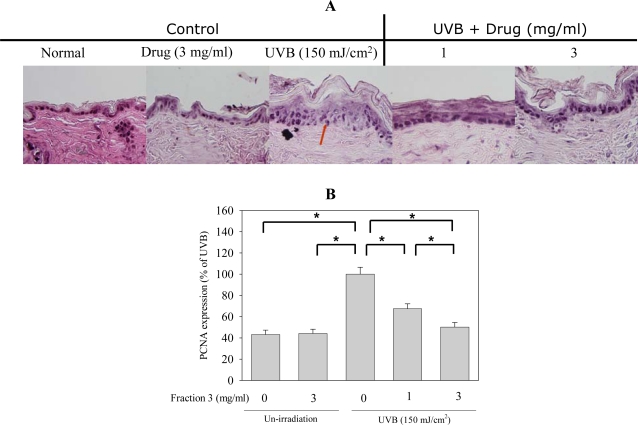
Fraction 3 inhibits UVB irradiation increased PCNA expression. (**A**) PCNA expression by immunohistochemistry, magnification 400 X. (**B**) Quantification of PCNA expression by counting positive staining cells, mean ± S.E. (n = 4). *p < 0.05 compared to the control group.

PCNA participates in the synthesis and metabolism of nucleic acid and plays an important role in DNA recombination, reproduction and repair. As DNA damage occurs, the expression of PCNA increases and executes the function to repair DNA. Therefore, PCNA expression is a marker of DNA repair and could be applied as an indicator of UVB-induced damage. As shown in Figure 7, after UVB exposure, the expression of PCNA increased ,which could be inhibited by pre-treatment with Fraction 3 (1 and 3 mg/mL) The PCNA expression decreased about 35 and 50% after treatment with 1 and 3 mg/mL of Fraction 3, respectively (Figure 7A and B). Thus, Fracton 3 could prevent the DNA damage by UVB exposure, which might contribute to decrease the PCNA expression [27].

## 3. Experimental Section

### 3.1. Chemicals

3-(4,5-dimethylthiazol-2-yl)-2,5-diphenyltetrazolium bromide (MTT), genistein, aprotinin, leupeptin, phenylmethylsulfonyl fluoride (PMSF), sodium fluoride (NaF), and sodium orthovanadate were purchased from Sigma Chemical. (St. Louis, MO). The antibody (Ab) raised against p-ERK1/2 was from Santa Cruz Biotechnology (Santa Cruz, CA). Abs raised against p38 and p-JNK were from Cell Signaling Technology (Beverly, MA). Abs raised against JNK, ERK1/2 and p-p38 were from R&D System. (Minneapolis, MN). The catalase assay kit was purchased from Molecular Probes (Eugene, OR). COX-2 and PCNA Abs were purchased from Lab Vision (Fremont, CA) and Santa Cruz Biotechnology, respectively.

### 3.2. Extraction of Soybean Isoflavones

Faction 3 (including aglycone and acetylglucoside form) was prepared by a method previously described by Kao *et al*. [8] with a minor modification. Briefly, a 50 g soybean cake sample was roasted for 20 min at 200 °C followed by mixing with 150 mL of ethanol/water (1:1 v/v) and shaken at room temperature for 2 h. Then the extracted sample was centrifuged at 6000 rpm for 20 min at 25 °C. The supernatant was collected and filtered through a glass filter paper. The soy isoflavones extract (80 mL) was poured onto a glass column (375 × 45 mm I.D.) containing Diaion HP-20 adsorbent (200 g) which was pre-activated with ethanol (1 L) and deionized water (1 L). The water-soluble impurities were eluted with deionized water (400 mL), followed by 15% aqueous ethanol (900 mL) to elute malonylglucosides and 27% aqueous ethanol (3300 mL) to elute glucosides. The residual isoflavones (Fraction 3) were eluted with 95% aqueous ethanol. The eluate was dried in a freeze-drying system and analyzed by high performance liquid chromatographic (HPLC) to evaluate the amount of each isoflavone. The concentration of each isoflavone in Fraction 3 is shown in Table 1. The isoflavone extract from soybean cake is water-soluble, so the tested isoflavones was dissolved in water for the *in vivo* and *in vitro* experiments.

### 3.3. Cell Viability Assay (MTT Assay)

Human immortalized keratinocytes (HaCaT cells) were maintained in Dulbecco’s modified Eagle’s medium (DMEM) with 10% fetal calf serum (GibcoBRL, Invitrogen Life Technologies, Carlsbad, CA), 100 units/mL penicillin, and 100 μg/mL streptomycin (Sigma). Briefly, vehicle-, Fraction 3-, pretreated cells were exposed to UVB and incubated for an additional 24 h. Cells were irradiated in a Bio-Sun system illuminator from VL (Vilber Lourmat, France) with a UV peak at 312 nm. The UVB irradiation dose was 50 mJ/cm^2^, which took approximately 44–48 s to administer (at an irradiance of 1.26–1.29 mW/cm^2^). After a brief wash with medium, MTT (0.5 mg/mL in DMEM) was used to quantify living metabolically active cells. Mitochondrial dehydrogenases metabolize MTT to a purple formazan dye, which was measured photometrically at 550 nm. Cell viability was proportional to the absorbance measured.

### 3.4. Western Blotting

The analysis of JNK, ERK and p38 was determined by Western blot as previously described [14,28].

### 3.5. Animals and Measurement of Physiological Skin Functions

Six-week-old male ICR-Foxn/*^nu^* mice were obtained from the National Laboratory Animal Center, Taipei, Taiwan. Mice were randomized into 5 groups for the experiments and then animals were housed eight per cage with controlled temperature (21–25 °C), humidity (60 ± 5%), and light (12/12 h light/dark cycle) for 1 week. During the week, animals were allowed free access to water and food.

ICR-Foxn/*^nu^* mouse dorsal skin was treated with different concentrations of the isoflavones extract Fraction 3 before 150 mJ/cm^2^ UVB irradiation [29,30] for 7 days. The TEWL and erythema were measured before the first UVB irradiation and 24 h after the UVB irradiation. The surface changes in the dorsal skin were recorded by photography. The level of TEWL and erythema, were regularly measured with MPA-580 (Courage & Khazaka, Cologne, Germany).

### 3.6. Histology and Immunostaining

Skin specimens were excised for histological examination. They were immediately fixed in 10% neutral buffered formaldehyde and frozen in optimal cutting temperature (OCT) compound (Thermo, Waltham, MA) for hematoxylin and eosin staining as well as immunohistochemistry. Vertical sections of 7-μm thickness were cut with a cryotome (Leica CM3050, Wetzlar, Germany). The following primary Abs were used: affinity-purified rabbit polyclonal COX-2 Ab and rabbit polyclonal PCNA Ab. All incubations with primary Abs were performed in blocking buffer overnight at 4 °C. After incubation with the primary Ab, samples were incubated with biotinylated secondary Abs (anti-rabbit IgG antibody,) for 2 h at RT. Images from immunostaining were obtained using an Olympus IX70 Inverted System microscope (Tokyo, Japan) and SPOT Cam software (Sterling Heights, MI). Quantification was made by counting the various positive cells in an arbitrarily selected field at 400× magnification and the thickness of the epidermis was quantified by Image J 1.3.4s.

### 3.7. Catalase Assay

Skin homogenates were centrifuged at 5000 rpm for 10 min, and the supernatant fractions were used. Catalase was analyzed according to the manufacturer’s protocol. Briefly, catalase first reacts with H_2_O_2_ to produce water and oxygen (O_2_). Next the Amplex Red reagent reacts with a 1:1 stoichiometry with any unreacted H_2_O_2_ in the presence of horseradish peroxidase (HRP) to produce the highly fluorescent oxidation product, resorufin. Therefore, as catalase activity increases, the resorufin signal decreases. The results are typically plotted by subtracting the observed fluorescence from that of a no-catalase control.

### 3.8. Statistical Analysis

Values are presented as a percentage of the control group and shown as the mean ± S.E. Student’s *t*-test was used to assess the statistical significance between the groups. We considered *p* values of <0.05 as statistically significant.

## 4. Conclusions

In summary, our results demonstrated that soy isoflavone extract Fraction 3 from soybean cake could prevent human keratinocyte apoptosis, attenuate the level of erythema and TEWL, reduce the epidermal thickness and increase the catalase activity and inhibit COX-2 and PCNA expression in response to UVB exposure. The results imply that Fraction 3 could decrease the UVB-induced oxidative stress, inflammation and skin cell damage. In addition, in comparison with soy isoflavone extract ISO-1 and genistein, Fraction 3 possessed the highest efficiency in reducing UVB-induced oxidative stress and preventing human keratinocyte death. Therefore, the soy isoflavone extract Fraction 3 is a superior anti-photoaging agent for skin care, and the advantages include the properties that it is non-toxic, easily accessible, economical, convenient and environmentally friendly.

## Figures and Tables

**Table 1.** The concentration of each isoflavone in Fraction 3.

	**Compound**	**Concentration (mg/g powder)**
Ac	acetyldaidzin	1.11
	acetylglycitin	0.06
	acetylgenistin	4.73

Ag	daidzein	0.68
	glycitein	0.18
	genistein	1.11

	Total	7.86
